# Blastocysts originated from oocytes with smooth endoplasmic reticulum aggregates have a reduced euploidy rate: a retrospective cohort study

**DOI:** 10.3389/fendo.2024.1425578

**Published:** 2024-09-30

**Authors:** Pengcheng Kong, Jiaping Pan, Shanshan Liang, Mingru Yin, Xiaoming Teng

**Affiliations:** ^1^ Center of Reproductive Medicine, Shanghai First Maternity and Infant Hospital, Tongji University School of Medicine, Shanghai, China; ^2^ Department of Assisted Reproduction, Shanghai 9th People’s Hospital Affiliated to Shanghai Jiaotong University School of Medicine, Shanghai, China

**Keywords:** oocyte morphology, smooth endoplasmic reticulum aggregates, blastocyst, preimplantation genetic testing, euploidy rate

## Abstract

**Research question:**

Does the presence of smooth endoplasmic reticulum aggregates (SERa) in oocytes adversely impact the euploidy rate of subsequent blastocysts?

**Design:**

We performed a retrospective cohort study with 671 young patients (< 38 years) undergoing their first preimplantation genetic testing for aneuploidy (PGT-A) between January 2019 and October 2022 at a reproductive medical center of university affiliated teaching hospitals in China. Cycles were categorized as either SERa(+) cycles (containing at least one SERa(+) oocyte) or SERa(-) cycles (all oocytes without SERa). In SERa(+) cycles, oocytes were further subdivided into the SERa(+) oocyte group and the sibling SERa(-) oocyte group, comprising oocytes with normal morphology.

**Results:**

No significant differences were observed in the normal fertilization rate (72.9% vs. 75.4% vs. 72.6%, P=0.343), and cleavage rate (96.8% vs. 97.1% vs. 96.4%, P=0.839) among the SERa(-) cycle group, the SERa(-) oocyte group, and the SERa(+) oocyte group. Additionally, there were no statistically significant differences in the rates of good quality embryos (44.7% vs. 48.8% vs. 46.2%, P=0.177) or blastocyst formation (60.1% vs. 60.9% vs. 60.5%, P=0.893) among the groups. However, the euploidy rate of blastocysts derived from SERa(+) oocytes was significantly lower compared to those from SERa(-) oocytes in SERa(+) cycles and normal oocytes in SERa(-) cycles (39.3% vs. 51.2% vs. 54.5%, P=0.005). Despite this, there were no significant differences in pregnancy and neonatal outcomes after euploid embryo transfer among the three groups.

**Conclusions:**

Blastocysts derived from SERa(+) oocytes have a lower euploidy rate than those derived from SERa(-) oocytes. Nevertheless, comparable reproductive outcomes were achieved following euploid embryo transfer from both SERa(+) and SERa(-) oocytes.

## Introduction

The quality of the oocyte is a crucial determinant for the potential subsequent embryo development and the success rate of *in vitro* fertilization (IVF) (1–3). Evaluating oocyte quality typically involves examining various morphological features, including the morphology of the first polar body, the size of the perivitelline space, defects in the zona pellucida, abnormal shapes, and the existence of refractile bodies, dense cellular granulation, vacuoles, and SERa (4). SERa, an abnormality characterized by multiple flattened, membrane-enclosed sacs or tubules of smooth endoplasmic reticulum surrounded by mitochondria, is observed under a scanning electron microscope (5). Under an inverted microscope, SERa differs from fluid-filled vacuoles in that it lacks fluid and is not separated from the cytoplasm by a membrane (6).

SERa is frequently observed in metaphase II (MII) oocytes, but its impact on IVF outcomes remains controversial. The Istanbul Consensus in 2011 discouraged the utilization of SERa(+) oocytes in IVF procedures due to previous research suggesting an increased risk of abnormalities in offspring derived from these oocytes (7–9). Additionally, several studies have reported declines in fertilization rates, poorer embryonic outcomes, and reduced pregnancy outcomes associated with SERa(+) oocytes or cycles (7, 8, 10, 11). However, other studies have found no evidence of abnormal embryonic outcomes from SERa(+) oocytes, nor decreases in pregnancy rates or adverse neonatal outcomes (12–14). The 2017 revision of the Alpha/ESHRE consensus suggested a case-by-case approach when dealing with SERa(+) oocytes (15). As a result, practices in assisted reproductive technology (ART) for managing SERa(+) oocytes vary widely due to the lack of clear guidelines. Given that embryo aneuploidy is a major cause of implantation failure, miscarriage, and birth defects (16), conducting PGT-A to select euploid embryos for transfer is more likely to result in a successful pregnancy and a healthy offspring (17). While several studies have suggested that the presence of SERa does not significantly affect the aneuploidy rate of subsequent blastocysts (18–20), these studies often did not account for the potential influence of male factors on embryo ploidy. Additionally, there is limited data on the safety of offspring born from SERa(+) oocytes.

In this study, we aimed to investigate whether the presence of SERa in oocytes adversely affects the ploidy of subsequent blastocysts in young women undergoing PGT-A cycles. This hypothesis is based on previous reports linking SERa with reduced pregnancy rates and higher miscarriage rates (7, 11). Furthermore, we evaluated the pregnancy and neonatal outcomes of transferred euploid blastocysts derived from SERa(+) oocytes, sibling SERa(-) oocytes in SERa(+) cycles, and blastocysts from SERa(-) cycles.

## Materials and methods

### Patients and study design

**Figure 1:**
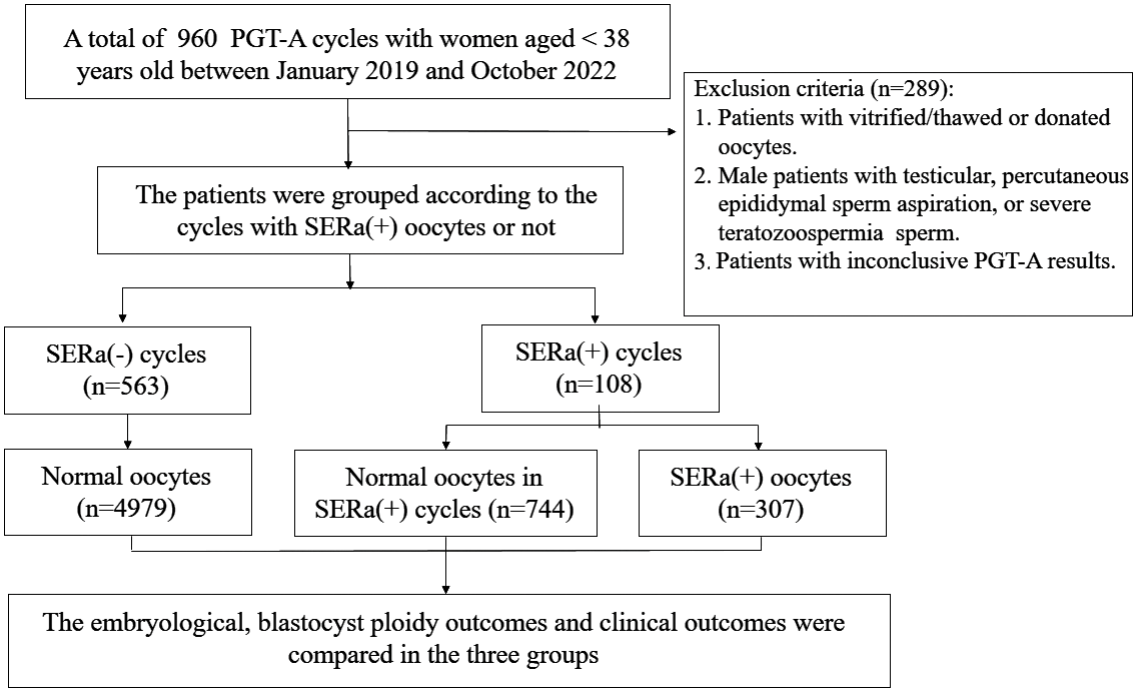
Flow chart of the study.

The retrospective cohort study included young women (aged <38 years) who underwent PGT-A at the assisted reproduction center, Shanghai First Maternity and Infant Hospital affiliated with Tongji University, between January 2019 and October 2022. The study was conducted and approved by the Ethical Committee of Shanghai First Maternity and Infant Health Hospital. The analysis did not include patients who were undergoing ICSI (intracytoplasmic sperm injection) with vitrified/thawed or donated oocytes, surgical sperm retrieval, severe teratozoospermia, preimplantation genetic testing for monogenic (PGT-M) or chromosomal structural rearrangements (PGT-SR). In addition, the analysis did not include embryos with inconclusive PGT-A results, indicating that there was insufficient DNA for testing. The flow chart of the inclusion of patients in this study is shown in **Figure 1**.

In this study, MII oocytes that had one or more visible SERa after cumulus cell denudation were classified as SERa(+) oocytes. The PGT-A cycles were divided into two categories: SERa(+) cycles, which included cycles with at least one SERa(+) oocyte, and SERa(-) cycles, which consisted of cycles with only SERa-free oocytes. The oocytes in the SERa(+) cycle groups were then categorized into two subgroups: the SERa(+) oocytes group and the sibling SERa(-) oocytes group, where the oocytes were morphologically normal.

### Oocyte preparation, observation, ICSI

All patients received standard protocols for controlled ovarian stimulation (COS) treatment. The details of the controlled ovarian hyperstimulation (COH) protocols have been previously established (21). Follicle development was monitored using transvaginal ultrasound. Recombinant human chorionic gonadotropin (HCG, Livzon, China) was administered as a trigger when two to three dominant follicles with diameters exceeding 18 mm were present.

**Figure 2:**
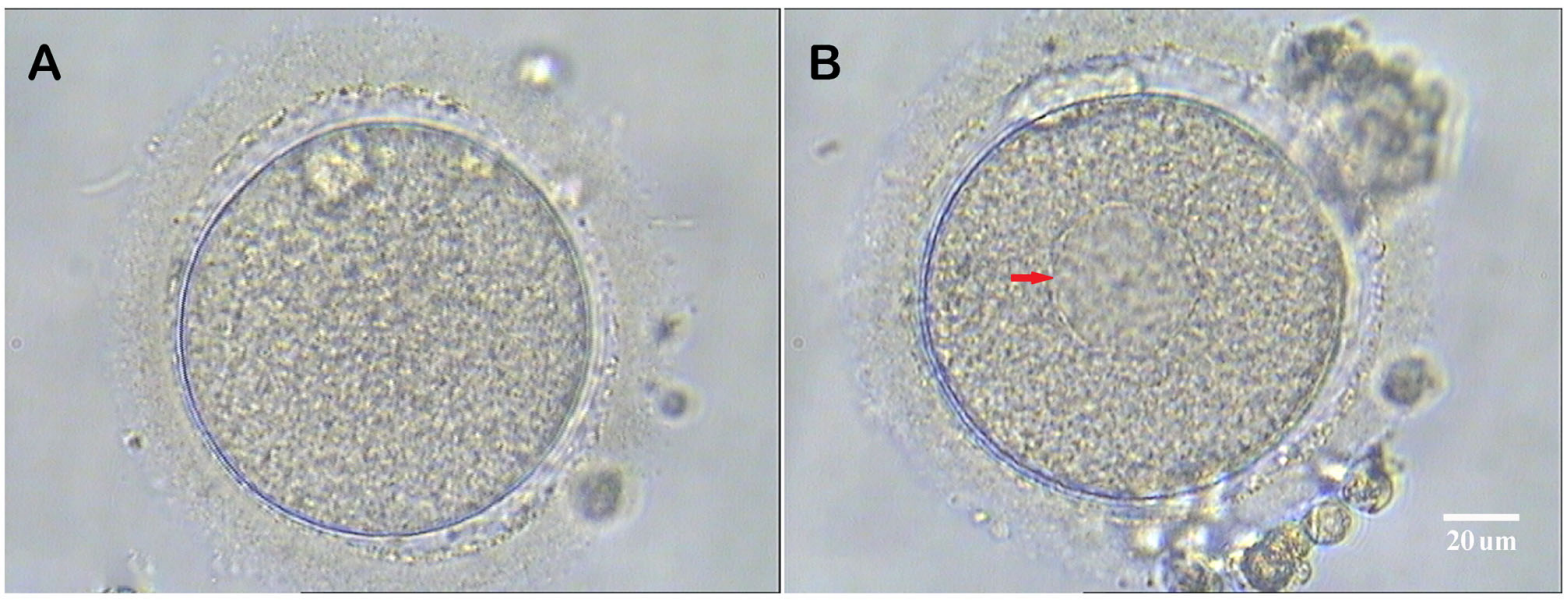
Human metaphase II oocytes (400×). **(A)** Normal metaphase II oocyte; **(B)** Metaphase II oocyte containing a smooth endoplasmic reticulum aggregates (red arrow). Scale bar = 20 μm.

Under ultrasound guidance, oocyte retrieval was conducted transvaginally 34-36 hours after administering the ovulatory trigger. The retrieved cumulus-oocyte complexes (COCs) were stored in the G-IVF PLUS medium (Vitrolife, Sweden) for 2 to 3 hours before removing the cumulus cells. The COCs were quickly blown and sucked several times in the HEPES-buffered medium containing hyaluronidase (80 IU/mL; LifeGlobal) using a Pasteur tube to remove the surrounding cumulus cells. The remaining cumulus cells were gently removed using a hand-drawn Pasteur pipette in MOPS PLUS medium (Vitrolife, Sweden). Oocyte morphology was examined at 400x magnification using an inverted Nikon Diaphot microscope equipped with a Hoffmann modulation contrast system, before ICSI. Only MII oocytes were utilized for ICSI, and those with SERa in the ooplasm were documented. At high magnifications, SERa in the cytoplasm presented as round, flat, and clear structures (**Figure 2**).

### Fertilization check and embryo quality assessment

The research exclusively employed ICSI technique for fertilizing the oocytes. On Day 1, the embryos were checked for fertilization. On Days 3, 5, and 6, the quality of the embryos was analyzed for morphological grading using the standard guidelines mentioned earlier (22). The system evaluates blastocyst quality based on three key features: Expansion Stage: Early Blastocyst (I, II): The blastocyst cavity is small or just beginning to expand; Expanding Blastocyst (III): The blastocyst cavity is expanding, but the blastocyst is not yet fully expanded; Expanded Blastocyst (IV): The blastocyst is fully expanded, with the cavity fully expanded and the zona pellucida stretched; Hatching Blastocyst (V, VI): The blastocyst has started to hatch or has completely hatched out of the zona pellucida. Inner Cell Mass (ICM): A (Good): A large number of cells are tightly packed; B (Fair): A moderate number of cells are loosely grouped; C (Poor): Very few cells are sparsely arranged. Trophectoderm (TE): A (Good): Cells are large and well-defined, with no or very few cells out of place; B (Fair): Cells are large and well-defined, but some cells are out of place; C (Poor): Cells are small and fragmented, with many cells out of place. Normal fertilization was defined as oocytes with two distinct pronuclei (2PN) and two polar bodies (2PB). The zygotes were cultured using sequential media, specifically G1-plus and G2-plus medium (Vitrolife, Sweden) until they reached the blastocyst stage on Day five or six.

### Blastocyst biopsy by NGS

Only high-quality blastocysts were chosen for biopsies to perform PGT-A analysis. The trophectoderm biopsy process involved securing the embryo with a holding pipette and then using laser pulses from the ZILOS-tk Laser to puncture the zona pellucida. Subsequently, a biopsy pipette with an internal diameter of 20 μm was used to extract a range of 3-5 cells. To obtain a sufficient amount of DNA for analysis, the Qiagen Multiple Displacement Amplification (MDA) DNA amplification system was utilized for whole genome amplification (WGA). Following the fragmentation of genomic DNA, PCR was employed to amplify and ligate adaptors. The Qubit dsDNA HS Assay kit (Life Technologies, Q32854) was utilized to quantify the libraries, and a final concentration of 250pM per library was loaded onto the PI chip for sequencing on the Ion Proton System (Ion PI Hi-Q Sequencing 200 Kit, Life Technologies, A26772). All sequencing operators have received standardized and unified operational training and hold certificates.

### Outcome assessments

The outcomes in the current study primarily included results from the IVF laboratory and clinical outcomes. The 2PN fertilization rate is determined by dividing the number of normally fertilized oocytes by the number of mature oocytes that were injected. The cleavage rate is defined as the ratio of cleaved fertilized oocytes to normally fertilized oocytes. The rate of good-quality embryos is defined as the number of good embryos divided by the number of normally fertilized oocytes. The blastocyst formation rate is calculated by dividing the number of blastocysts formed by the number of normally fertilized oocytes. Good-quality blastocysts were identified as those assigned a score of 3BC or higher according to the Gardner scoring system.

An elevated serum HCG level was recorded as hCG-positive. Clinical pregnancy is defined as the detection of fetal heart activity in gestational sacs using ultrasound six weeks after embryo transfer. The miscarriage rate was calculated by dividing the number of miscarriages before the 20th week by the number of women who had a clinical pregnancy. Live birth was defined as the delivery of a live infant at a gestational age of 28 weeks or more.

### Statistical analysis

The one-sample Kolmogorov-Smirnov test was used to assess the normal distribution of continuous data. Continuous variables were reported as mean ± SD when regularly distributed. The differences in the continuous variables were analyzed using the t-test. The categorical variables were presented as percentages, and the differences are analyzed using the chi-squared test or Fisher’s exact test, as appropriate. General Linear Model analysis was carried out to survey the relationship between the presence of SERa and blastocyst euploidy rate with adjustment for possible confounding factors. The Statistical Program for Social Sciences (SPSS Inc., Version 22.0, Chicago, IL, USA) was used for the statistical analysis. A *p*-value less than 0.05 in a two-tailed test is considered statistically significant.

## Results

A total of 671 PGT-A cycles were included in this study, of which 108 cycles had at least one oocyte with SERa. For the control group, 563 PGT-A cycles without SERa(+) oocytes were included during the same period. As summarized in **Table 1**, there were no significant differences between the SERa(+) and SERa(-) cycle groups in terms of mean female age, mean male age, mean female body mass index (BMI), infertility types, stimulation protocols, basal follicle-stimulating hormone (FSH) levels, dosage and duration of gonadotropin administration, estradiol levels on the day of hCG trigger, mean number of retrieved oocytes, or mean number of metaphase II (MII) oocytes.

**Table 1:** Baseline characteristics of patients in study groups.

	SERa (-) cycles (n= 563)	SERa (+) cycles (n=108)	*P*
Number of cycles	563	108	
Female age (years)	32.6± 3.1	32.2 ± 3.5	0.266
Male age (years)	34.2± 4.4	33.6± 3.9	0.145
Female BMI (kg/m2)	21.8± 2.8	21.8± 3.0	0.849
Infertility types			0.523
Primary, % (n)	41.6 (234/563)	38.0 (41/108)	0.523
Secondary, % (n)	58.4 (329/563)	62.0 (67/108)	0.523
COH protocols			0.821
GnRH agonist protocol, % (n)	53.6 (302/563)	51.9 (56/108)	0.753
GnRH antagonist protocol, % (n)	31.3 (176/563)	34.3 (37/108)	0.573
Other protocol, % (n)	15.1 (85/563)	13.9 (15/108)	0.772
Duration of infertility (years)	2.4± 2.4	2.1± 2.3	0.239
Basal FSH level (IU/L)	7.1± 2.8	7.3± 2.9	0.599
Total FSH dosage (IU)	2020.1 ± 860.6	2130.4 ± 980.4	0.234
Days of stimulation	9.4 ± 2.9	9.9 ± 2.9	0.122
Estradiol on hCG day (pg/mL)	3157.9 ± 1627.3	3064.8 ± 2153.6	0.739
P on day of hCG (ng/mL)	1.1 ± 0.7	1.1 ± 0.6	0.400
Mean no. of oocytes retrieved	11.3 ± 6.9	12.2 ± 6.7	0.210
Mean no. of MII oocytes	8.8 ± 5.7	9.7 ± 5.6	0.136

Data are presented as mean ± standard deviation for continuous variables and % (n) for categorical variables. COH, controlled ovarian hyperstimulation; GnRH, gonadotropin-releasing hormone; FSH, follicle-stimulating hormone; P, progesterone. The differences in the continuous variables were analyzed using the t-test. The categorical variables were presented as percentages, and the differences are analyzed using the chi-squared test.

In terms of embryological outcomes, as shown in **Table 2**, there were 307 SERa(+) oocytes and 744 SERa(-) oocytes in the SERa(+) cycles. The main embryological outcomes were compared between normal oocytes (n=4979) in the SERa(-) cycles, SERa(-) oocytes (n=744), and sibling SERa(+) oocytes (n=307) in SERa(+) cycles. No statistically significant differences were found in the normal fertilization rate (72.9% vs. 75.4% vs. 72.6%, respectively, *P*=0.343), abnormal fertilization rate (3.3% vs. 4.7% vs. 4.9%, respectively, *P*=0.07), or cleavage rate (96.8% vs. 97.1% vs. 96.4%, respectively, *P*=0.839) among the groups. Similarly, there were no significant differences in the rate of good-quality embryos (44.7% vs. 48.8% vs. 46.2%, respectively, *P*=0.177) or blastocyst formation rate (60.9% vs. 60.1% vs. 60.5%, respectively, *P*=0.893). We also examined the euploidy rate of high-quality blastocysts formed from SERa(+) oocytes and sibling SERa(-) oocytes, and normal oocytes from SERa(-) cycles, as shown in **Table 2**. Notably, the euploidy rate of blastocysts derived from SERa(+) oocytes was significantly lower than that of blastocysts from sibling SERa(-) oocytes and normal oocytes from SERa(-) cycles (39.3% vs. 51.2% vs. 54.5%, *P* = 0.005). After adjusting for several potential confounders, including female age, BMI, basal FSH levels, and basal progesterone (P) levels, a negative association between the presence of SERa and the euploidy rate remained in the Generalized Linear Model (β = -0.165, SE = 0.05, P = 0.001), as shown in **Table 3**. Female age was also found to be an independent predictor of the blastocyst euploidy rate (P < 0.001).

**Table 2:** Embryological and blastocyst ploidy outcomes among the groups.

	Oocytes in SERa (-) cycles	SERa (-) oocytes in SERa (+) cycles	SERa (+) oocytes in SERa (+) cycles	*P*
Normal fertilization rate, % (n)	72.9 (3629/4979)	75.4 (561/744)	72.6 (223/307)	0.343
Abnormal fertilization rate, % (n)	3.3 (165/4979)	4.7 (35/744)	4.9 (15/307)	0.070
Cleavage rate, % (n)	96.8 (3512/3629)	97.1 (545/561)	96.4 (215/223)	0.839
Good-quality embryo rate, % (n)	44.7 (1622/3629)	48.8 (274/561)	46.2 (103/223)	0.177
Blastocyst formation rate, % (n)	60.1 (2176/3629)	60.9 (342/561)	60.5 (135/223)	0.893
Euploidy rate, % (n)	54.5 (914/1676)^a^	51.2 (155/303) ^a^	39.3 (44/112)^b^	0.005^*^

*P < 0.05 was considered statistically significant. The differences are analyzed using the chi-squared test. Values with different superscript letters within each column are significantly different (P < 0.05).

The bold values indicate significant differences.

**Table 3:** General Linear Model (GLM) analysis of blastocyst euploidy rate among participants.

Model	B	SE	*P*
Group 2	-0.165	0.050	0.001
Group 1 (reference)			
Female age (years)	-0.037	0.008	<0.001
Female BMI (kg/m2)	-0.002	0.007	0.768
Basal FSH level (IU/L)	-0.004	0.007	0.536
Basal P level (ng/mL)	0.028	0.033	0.394

SE, standard error; BMI, body mass index; Group1, SERa(-) cycles; Group2, SERa(+) cycles. GLM: euploidy rate by groups with female age, female BMI, basal FSH level, basal P level.

At our center, only one embryo is transferred per PGT-FET cycle. Clinical and neonatal outcomes for the three groups were presented in **Table 4**. A total of 358 euploid blastocysts were transferred in the SERa(-) cycles, while 82 euploid blastocysts were transferred in the SERa(+) cycles, 19 of which were derived from SERa(+) oocytes. There were no significant differences in the hCG-positive rate, clinical pregnancy rate, or miscarriage rate among blastocysts obtained from SERa(+)oocytes, sibling SERa(-) oocytes, and normal oocytes from SERa(-) cycles. Although the live birth rate in the SERa(+) oocyte group was slightly lower than that in the other two groups, the difference was not statistically significant. Neonatal outcomes were similar across all three groups, and no birth defects were reported in newborns derived from SERa(+) oocytes.

**Table 4:** Clinical and neonatal outcomes in PGT-FET cycles among the groups.

	Oocytes in SERa (-) cycles	SERa (-) oocytes in SERa (+) cycles	SERa (+) oocytes in SERa (+) cycles	*P*
Clinical outcomes				
No. of transferred cycles	358	63	19	
hCG positive rate, % (n)	61.2 (219/358)	60.3 (38/63)	57.9 (11/19)	0.959
Clinical pregnancy rate, % (n)	59.5 (213/358)	58.7 (37/63)	52.6 (10/19)	0.864
Miscarriage rate, % (n)	6.7 (24/358)	9.5 (6/63)	10.5 (2/19)	0.597
Neonatal outcomes				
Live birth rate, % (n)	52.8 (189/358)	49.2 (31/63)	42.1 (8/19)	0.618
Gestational age (weeks)	38.0 ± 2.0	38.5 ± 0.9	38.9 ± 0.6	0.183
Birth weight (g)	3312 ± 566.1	3382 ± 448.3	3239 ± 265.5	0.733
Average length (cm)	49.9 ± 2.3	49.9 ± 2.0	50.0 ± 0.9	0.988
Congenital abnormalities rate, % (n)	3.2 (6/189)	3.2 (1/31)	0 (0/8)	1.000

Data are presented as mean ± standard deviation for continuous variables and % (n) for categorical variables. The differences are analyzed using the chi-squared test or Fisher’s exact test, as appropriate.

## Discussion

In this study, we found that while blastocyst formation rates were comparable between SERa(+) oocytes and SERa(-) oocytes, blastocysts derived from SERa(+) oocytes exhibited significantly lower euploidy rates than those from SERa(-) oocytes. However, our findings also indicated that euploid blastocysts derived from SERa(+) oocytes can achieve pregnancy outcomes comparable to those derived from SERa(-) oocytes. Moreover, blastocysts from SERa(+) oocytes can lead to the birth of healthy children, suggesting that euploid blastocysts from SERa(+) oocytes are suitable for transfer.

The role of calcium ion (Ca²^+^) accumulation and release by the endoplasmic reticulum (ER) is critical during oocyte development and fertilization (23). In most species, the ER forms distinct clusters in the cortical ooplasm as the oocyte matures. In contrast, SERa(+) oocytes exhibit a distinct morphological appearance, appearing as visible round vesicles containing numerous elongated, curved, dense tubular smooth endoplasmic reticulum (5). This cytoplasmic abnormality may disrupt calcium storage and oscillations during fertilization. Several studies have reported a significant decrease in fertilization rates in cycles with SERa(+) oocytes (5, 24). Nevertheless, other studies, including ours, have found no significant differences in fertilization rates between SERa(+) and SERa(-) oocytes (10, 11, 25). Our results also showed that while the abnormal fertilization rate was slightly higher in SERa(+) oocytes, this difference was not statistically significant.

Conflicting results exist regarding the impact of SERa on embryonic outcomes. Some studies have reported lower blastocyst formation rates in SERa(+) oocyte cycles (5, 8), with a significantly reduced proportion of good-quality blastocysts derived from SERa(+) oocytes (1, 14). Conversely, other studies found no impact of SERa on blastocyst formation rates (11, 26). Our findings align with these latter studies, as we observed no significant difference in the rates of good-quality embryos or blastocyst formation between SERa(+) and SERa(-) oocytes. The presence of SERa in oocytes has been linked to negative effects on blastocyst quality and developmental speed (27). Braga et al. (1) reported a significant decrease in pregnancy success rates and an elevated risk of miscarriage in cycles with SERa(+) oocytes. Despite similar fertilization and early embryo development rates, embryos from SERa(+) oocytes have been shown to exhibit lower implantation rates (11). Consistent with these findings, our study revealed a reduced blastocyst euploidy rate in SERa(+) oocytes compared to SERa(-) oocytes. This lower euploidy rate could explain the previously reported decreased clinical pregnancy rates and elevated miscarriage rates associated with SERa(+) cycles (7, 9, 26). Although it has been proposed that the blastocyst euploidy rate in SERa(+) cycles may remain unaffected (19), limitations such as the small sample size of the SERa(+) group reduce the number of blastocysts available for euploid analysis. Additionally, previous research did not utilize multivariate analysis to control for confounding factors such as female age, ovarian response and male factors, which could influence the results (18, 20, 28).

The smooth endoplasmic reticulum (SER) plays a crucial role in regulating intracellular calcium homeostasis, essential for oocyte maturation, spindle formation, and chromosome segregation (29). SER aggregates may disrupt this calcium balance, potentially leading to abnormal calcium signaling during meiosis and errors in chromosome segregation. Previous research has shown that SERa(+) oocytes exhibit intrinsic cytoskeletal damage, including spindle size alterations, chromosome misalignment, and cortical actin disorganization (30). Additionally, gene expression profiles of SERa(+) oocytes differ significantly from those of SERa (–) oocytes, showing downregulation of genes involved in cell division, spindle assembly, cytoskeletal organization, and mitochondrial activity (31). These molecular disruptions suggest that SER aggregates can impair essential cellular processes in oocytes, thereby increasing the risk of meiotic errors and ultimately reducing euploidy rates. While further mechanistic studies are needed to definitively verify the relationship between SERa(+) oocytes and embryo euploidy rates, current evidence strongly suggests a potential link between SER aggregates and impaired oocyte quality.

The discrepancies between our findings and those of earlier research may be due to two major factors. First, the heterogeneity in the formation of SERa(+) oocytes could contribute to this inconsistencies. Research has indicated that the presence of SERa correlates with increased serum progesterone levels, often resulting from prolonged ovarian stimulation or administration of high doses of gonadotropins (8, 26). It has been suggested that the occurrence of SERa is more closely related to elevated progesterone levels than to estradiol levels (32). One study found that no SERa(+) oocytes were observed in unstimulated patients, suggesting that the occurrence of SERa may be related to the use of exogenous gonadotropin stimulation (33). Conversely, recurrent cases of SERa in the same individual across multiple ovarian stimulation cycles suggest a possible genetic predisposition (9, 34). This significant diversity in the composition and formation of SERa(+) oocytes may explain the contradictory clinical outcomes reported, as embryos derived from SERa(+) oocytes could exhibit varying developmental and pregnancy success. Second, the detection of SERa can be influenced by factors such as embryologist experience, training, and documentation practices, leading to variability in reported rates. Additionally, differences in study design, sample size, ovarian stimulation protocols, and patient populations can contribute to divergent outcomes. These factors may introduce biases affecting embryonic development and pregnancy outcomes. The reliance on single-center studies in current SERa-related research limits generalizability. To mitigate potential biases, this study employed two experienced embryologists for observation and documentation, ensuring reliability. Furthermore, cases with abnormal sperm morphology were excluded, minimizing paternal influence on embryo euploidy, a confounding factor not addressed in prior studies. Despite these efforts, large-scale, prospective, multicenter studies are needed to clarify the relationship between SERa and embryonic euploidy.

Previous studies have suggested a higher incidence of birth defects following the transfer of embryos from SERa(+) oocytes (7–9). However, more recent studies have reported the birth of healthy babies from SERa(+) oocytes (12, 13). Our findings are consistent with these reports, showing no significant differences in pregnancy outcomes or neonatal malformations when transferring euploid embryos from SERa(+) oocytes compared to SERa(-) oocytes. The small number of transferred embryos from SERa(+) oocytes in our study, however, may have reduced statistical power, increasing the risk of Type II errors. Although appropriate statistical methods were applied, the small sample size limits the ability to detect potential differences. Future studies should increase sample sizes to further investigate these differences.

This study has several limitations. As a retrospective study, it is subject to inherent biases and cannot control for participant heterogeneity. Additionally, the number of offspring born from SERa(+) oocytes is relatively small, necessitating cautious interpretation of the results. Future research with larger sample sizes and prospective designs is essential to validate our findings. Long-term follow-ups on newborns from SERa(+) oocytes are also needed to assess the possibility of developmental abnormalities.

In summary, this study provides evidence of a negative correlation between SERa(+) oocytes and embryonic euploidy, suggesting that the presence of SERa may serve as a diagnostic marker for identifying embryos at higher risk of chromosomal abnormalities in assisted reproductive techniques (ART). By identifying oocytes with SER aggregates, clinicians may prioritize embryos from SERa(-) oocytes for transfer, potentially improving success rates and reducing miscarriage risks. Further research is needed to validate this approach and refine the diagnostic criteria for using SERa as a predictive marker, but these findings offer promise for optimizing embryo selection and improving ART outcomes.

## Data Availability

The original contributions presented in the study are included in the article/supplementary material. Further inquiries can be directed to the corresponding authors.
